# The Effect of Oxytocin on Social and Non-Social Behaviour and Striatal Protein Expression in C57BL/6N Mice

**DOI:** 10.1371/journal.pone.0145638

**Published:** 2015-12-30

**Authors:** Xiaofan Zhang, Qi Li, Min Zhang, Sylvia Lam, Pak Chung Sham, Bitao Bu, Siew Eng Chua, Wei Wang, Grainne Mary McAlonan

**Affiliations:** 1 Department of Neurology, Tongji Hospital, Tongji Medical College, Huazhong University of Science and Technology (HUST), 1095 Jiefang Ave., Wuhan, 430030, P.R. China; 2 Department of Psychiatry, University of Hong Kong, Pokfulam, Hong Kong Special Administrative Region (H.K.S.A.R.), China; 3 Department of Forensic and Neurodevelopmental Sciences, The Institute of Psychiatry, Psychology and Neuroscience, King’s College London, London, United Kingdom; CNRS UMR7275, FRANCE

## Abstract

Oxytocin has been suggested as a promising new treatment for neurodevelopmental disorders. However, important gaps remain in our understanding of its mode of action, in particular, to what extent oxytocin modulates social and non-social behaviours and whether its effects are generalizable across both sexes. Here we investigated the effects of a range of oxytocin doses on social and non-social behaviours in C57BL/6N mice of both sexes. As the striatum modulates social and non-social behaviours, and is implicated in neurodevelopmental disorders, we also conducted a pilot exploration of changes in striatal protein expression elicited by oxytocin. Oxytocin increased prepulse inhibition of startle but attenuated the recognition memory in male C57BL/6N mice. It increased social interaction time and suppressed the amphetamine locomotor response in both sexes. The striatum proteome following oxytocin exposure could be clearly discriminated from saline controls. With the caveat that these results are preliminary, oxytocin appeared to alter individual protein expression in directions similar to conventional anti-psychotics. The proteins affected by oxytocin could be broadly categorized as those that modulate glutamatergic, GABAergic or dopaminergic signalling and those that mediate cytoskeleton dynamics. Our results here encourage further research into the clinical application of this peptide hormone, which may potentially extend treatment options across a spectrum of neurodevelopmental conditions.

## Introduction

Oxytocin has a recognised role in lactation and parturition. However, it also has central binding sites in the limbic system and basal ganglia [1] and is now appreciated to be involved in the regulation of a wide variety of social and non-social behaviours [2]. As a consequence, oxytocin has been proposed to have utility in neurodevelopmental disorders of social processing and cognition. Emerging evidence suggests oxytocin can serve as an antipsychotic that modulates glutamatergic signalling [3]. Indeed, several studies have reported that oxytocin ameliorated symptoms of schizophrenia [4] and exerted a generally positive impact on social behaviour, cognition and memory in humans [5].

Preclinical studies are broadly in agreement. Social interaction deficits in rats caused by chronic phencyclidine administration were reversed by oxytocin [6] and rats treated with anti-psychotics had elevated oxytocin secretion, suggesting that endogenous oxytocin contributes to the antipsychotic action of conventional antipsychotic drugs [3]. However, there remain a number of gaps in our understanding of oxytocin’s therapeutic potential. First, pre-clinical studies often examine a limited range of behavioural tasks, and it is unclear whether oxytocin modulates both social and non-social behaviours at the doses used. Second, many studies have a male bias but, since brain oxytocin and oxytocin receptor distributions are sex specific [7], it is not known if results generalize across both sexes. Third, the protein substrates in the neural networks targeted by oxytocin remain relatively obscure. Therefore, in this study we sought to clarify the effects of a range of oxytocin doses on social and non-social behaviours in female and male C57BL/6N mice. We hypothesized that peripherally injected oxytocin would improve performance in social and non-social tasks and alter protein expression in a similar direction to that reported for anti-psychotic medication.

We also predicted that oxytocin would alter protein expression in brain. We selected the striatum as a region-of -interest because it is a major neural substrate of oxytocin [8]; it is strongly implicated in neurodevelopmental disorders [9], linked to both social and non-social behaviour traits in these conditions [10]. For example, neuroanatomical differences relative to typically developing controls are consistently reported in autism and schizophrenia [11–14]. In line with this, preclinical studies have linked the striatum to behaviors relevant to these disorders including sensorimotor gating, social interaction and amphetamine sensitivity [15–21]. In addition, the striatum is considered to be a key target for the anti-psychotic action of medications used in schizophrenia [22–27]. Therefore, we predicted that oxytocin would alter striatal protein expression a similar direction to that reported in preclinical and clinical studies of conventional anti-psychotic treatment.

## Materials and Methods

### Animals

One hundred and sixteen adult C57BL/6N mice (8 weeks old, female = 58, male = 58) were used in this study of oxytocin. All mice were obtained from and housed in the Laboratory Animal Unit (LAU) at the University of Hong Kong. The experimental protocol had been approved by the Committee on the Use of Live Animals for Teaching and Research, the University of Hong Kong (CULATR case no: 2189–10, 2624–12). The behaviour holding room was maintained at 21°C but, unlike the general areas of the LAU, had a reversed day-night cycle (light on: 7PM-7AM). Mice were therefore acclimatized for a minimum of one week before testing. All behavioural tests were conducted in the dark phase of the light-dark cycle.

### Behavioural testing

#### Prepulse inhibition test (PPI)

The whole-body startle responses of mice were measured in mouse startle chambers supplied by SR-LAB (San Diego Instruments, San Diego, CA, USA). Mice were placed in the startle chamber immediately after either oxytocin or saline injection and left undisturbed for 10min before the PPI test began [28]. The PPI paradigm conducted followed the standard protocol in our lab [29]. As pharmacological treatments can alter baseline reactivity to startle stimuli, and confound interpretation of the PPI [30], for this paradigm we employed a design incorporating 3 prepulse and 3 pulse conditions [31]. This allowed us to exclude conditions where altered baseline reactivity might confound interpretation of PPI. All startle stimuli and background stimuli (NS) comprised white noise. The background stimulus was 65dB. The three intensities of a 40ms pulse were 100dB, 110dB and 120dB; the three 20ms prepulse intensities were 6dB, 12dB or 18dB above background. The session began with six consecutive pulse-alone trials. The first block (first six trials) had two of each pulse-alone condition to habituate and stabilize the startle response. These trials were not included in the statistical analysis. Thereafter the test session included 10 blocks of 16 trials in pseudorandom order. Each block was composed of three prepulse-alone trials (+6, +12 or +18dB), three pulse-alone trials (100, 110 or 120dB), 9 combinations of prepulse-pulse trials (3 prepulse options×3 pulse options), and one no-stimulus (NS) trial. The test session ended with a final block of six pulse-alone trials as in the first block. The whole session lasted approximately 45 minutes.

PPI data were collected by SR-LAB. PPI was taken as the reduction in pulse-induced startle reaction on prepulse_pulse trials relative to the reaction in the pulse_alone trials. The proportional (percentage) reduction of the startle amplitude (%PPI) based on the non-transformed startle response was calculated by the formula [(Rpulse_alone—Rprepulse_pulse)/ Rpulse_ alone] × 100%, where “R” was the individual mean response of the startle reaction. PPI data were analysed by repeated measures ANOVA, with pulse intensity (100 110 or 120 dB) and prepulse intensity (+6, +12 or +18 dB) as within-subject factors, and drug dose (saline and three doses of oxytocin), sex and drug exposure (single or repeated dose), as between-subject factors. Data are presented as (Mean ± SEM) and were analysed with IBM SPSS 22.0 software. P values less than 0.05 were considered to indicate statistical significance.

#### Novel object recognition test

**Fig 1:**
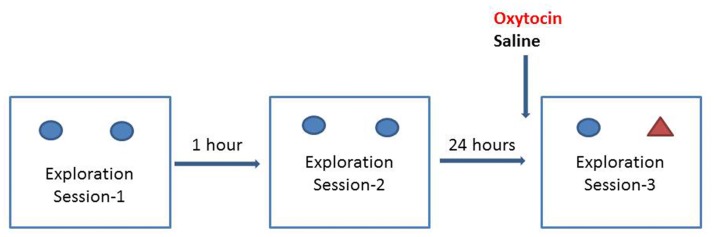
Novel object recognition test. Blue circles: A1, A2; original objects. Red triangle: B; novel object. Oxytocin doses: 10 μg/kg, 100 μg/kg and 1000 μg/kg. In the first day of the test, the subject explored the two objects of same colour and same shape twice in 1 hour interval. Oxytocin was administrated in the second test day, then the subject explored one of the same objects previously presented and a new object of different colour and shape.

Object recognition in mice followed a standard protocol [32]. Mice were placed into an open field (40 cm×40 cm) test box, in which two identical solid impermeable objects (A1, A2) were positioned at one end of the field, each 10cm from the adjacent walls. Mice were allowed to explore the area for 3mins (Exploration Session-1). One hour later, they were exposed to the same open field and objects, which had been thoroughly cleaned with 70% ethanol, for 3 mins (Exploration Session-2). Either oxytocin or saline was administered immediately after the second exploration session. After 24 hours, the mice were again exposed to the test apparatus this time containing one of the original objects (A2) and a novel object (B) (different size, shape and colour, Fig 1). The time spent exploring each of the objects (Exploration Session-3) was assessed over 3 min.

A discrimination index was calculated as the (exploration of novel object-exploration of familiar object)/ (total exploration time), i.e. (B-A2)/ (B+A2). All the mice had received one previous dose of drug injection respectively before the novel object recognition test. Recognition memory was assessed by the discrimination index. The novelty discrimination index was analysed using a two-way ANOVA, SPSS22, with sex and drug dose as between-subjects factors.

### Social interaction test

The social interaction test was carried out in a neutral cage with standard bedding (30 cm×30 cm×30 cm) following methods previously published [33]. On the first three days before the test, each mouse, including mice of the same strain, age and sex to be used as ‘strangers’, were habituated to the cage in one 10min session each day. On day 4, the test day, either oxytocin or saline was administered 30min before the test. Pairs of an unfamiliar, ‘stranger’ mouse and experimental mouse were placed into the neutral cage for 10 min. The time spent in social interaction (active contact such as sniffing, following and grooming the partner) was measured over 10 min.

The effects of oxytocin dose and exposure were analysed using a Univariate General Linear Model (GLM, SPSS 22), in which the drug dose and exposure (single or repeated) were the between-subject factors.

### Open field and amphetamine test

**Fig 2:**
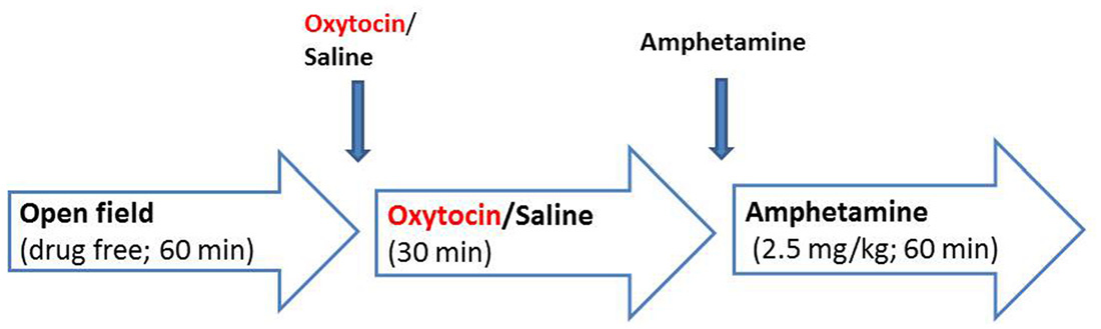
Open field and Amphetamine test. Oxytocin doses: 10 μg/kg, 100 μg/kg and 1000 μg/kg. The subject explored the arena for 1 hour while drug free. Then the subject explored the same arena for another 30min after drug (saline or oxytocin) injection. Finally the subject explored the same arena again for 1hour after amphetamine injection.

The open field exploration test was tested following methods previously published [34]. It was performed in 40×40 cm^2^ rectangular white arenas. At the beginning of the session, the mouse was gently placed in the centre of the arena, and allowed to explore for 1hour while drug free. The mouse was then briefly removed from the arena, and drug administered quickly via the subcutaneous (s.c.) route. The arena was cleaned with 70% ethanol; the mouse was placed back in the arena and allowed to explore for another 30min. The mouse was again briefly removed from the arena and injected with 2.5 mg/kg d-amphetamine (calculated as the salt, sigma-Aldrich, Switzerland) dissolved in a 0.9% NaCl solution via the intraperitoneal route. The volume of injection was 5 ml/kg. The arena was cleaned again. The mouse was placed back in the arena and allowed to explore for another 1hour (Fig 2).

### Order of behavioural testing

**Fig 3:**
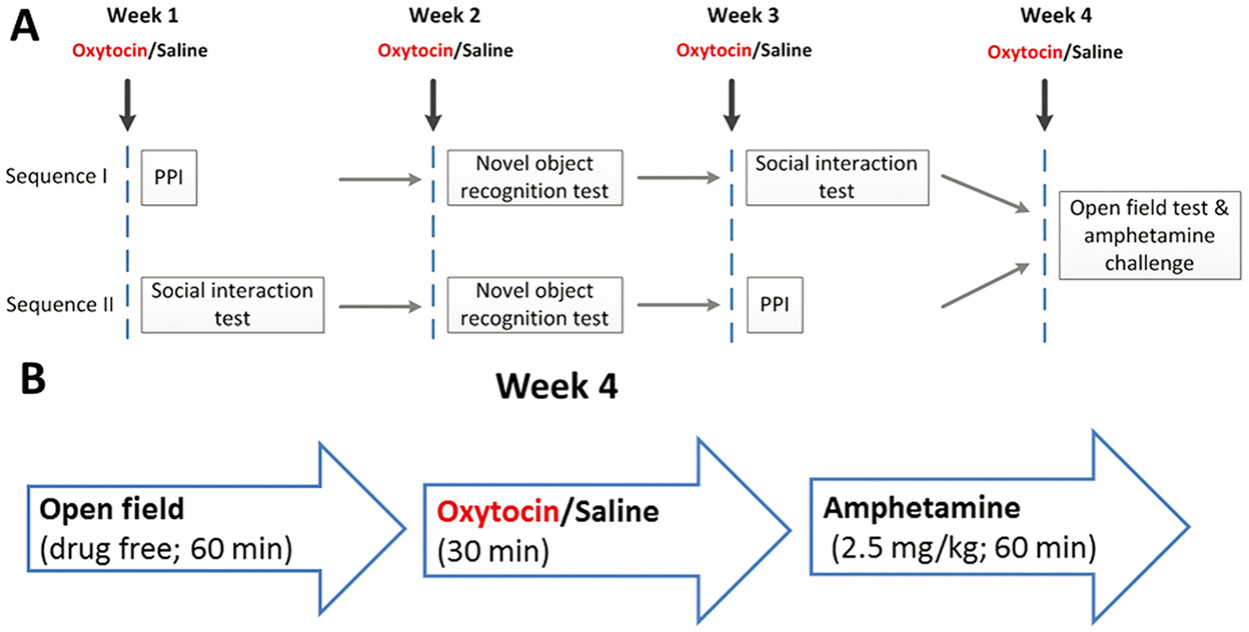
Behavioural tests. The sequence of behavioural testing. Mice were dived into two groups of equal number and sex. Half the mice followed test sequence I; half followed test sequence II. PPI: Prepulse inhibition of startle.

Equal numbers of male and female mice received a single subcutaneous dose of 10 μg/kg, 100 μg/kg or 1000 μg/kg of oxytocin (Sigma Chemicals, St Louis, Mo., USA) in an injection volume of 5 ml/kg prior to testing. All mice were given one week of ‘rest’ between each test. Each mouse received the same dose of oxytocin or saline for all tests. To control for the potential effect of previous oxytocin exposure and/or behavioural test order, half the mice followed test sequence I; half followed test sequence II (Fig 3). Twenty mice (10 females; 10 males) received saline injections only. There were 32 mice (16 females; 16 males) in each oxytocin dose group: 10 μg/kg, 100 μg/kg or 1000 μg/kg. Where appropriate, whether this was the first exposure to oxytocin or a repeat exposure was entered as a fixed factor in the analyses.

The open-field and amphetamine challenge test were carried out last; therefore, all animals had previously received 3 doses of oxytocin or saline. In this test, to clarify whether pre-exposure to oxytocin was sufficient to modify low-dose (2.5 mg/kg) amphetamine response, or whether an additional dose of oxytocin was necessary, half the cohort received saline immediately before the amphetamine challenge; half received a single dose of oxytocin (10 μg/kg, 100 μg/kg or 1000 μg/kg)

### Proteomics and Western blot

Twelve mice (female = 6, male = 6) were naïve to behavioural testing; half were exposed to 1000 μg/kg oxytocin, half to saline vehicle injection, once a week for 3 weeks were selected for proteomics and western blot analysis. The mice were sacrificed by cervical dislocation. The brain was removed and placed immediately on ice, and the striatum dissected free of the remainder of the brain tissue. The striatal tissue from each mouse was homogenized in 0.5ml of ice-cold 0.32 M sucrose buffered with 5 mM HEPES, pH7.5.

### Proteomics analyses

Striatum was homogenized in 2D lysis buffer (7M Urea, 2M Thiourea, 2% CHAPS, 10mmol DTT, 0.5% IPG buffer) for 30 seconds, then centrifuged at 15,000×g for 15 minutes to remove cell debris. Supernatant for 2-DE was collected and quantified with protein assay kit (Pierce^®^ 660nm Protein Assay, Thermo Scientific).

The 2D procedure followed the protocol from Bio-Rad Laboratories (Bio-Rad Laboratories, Inc.), with minor modifications. Rehydration and first dimension were carried out using Bio-Rad isoelectric focusing (IEF) separation (Bio-Rad IEF system). The 350ul rehydration buffer containing 180ug protein was loaded on 18cm, pH 4–7, IPG DryStrips (Bio-rad). IEF conditions were: 50V, 18h, 1000V, 1h; 3000V, 3h, 8000V, 6h. After the IEF run was complete, the IPG strips were equilibrated for 2×20min in the equilibration buffer containing 50mM Tris, 6M urea, 30% glycerol, 2% SDS. The first equilibration was performed in the equilibration buffer with 1.0%w/v DTT followed by a second equilibration with 2.5% w/v iodoacetamide. The strips were subsequently subjected to a secondary dimensional separation by 12% SDS-PAGE (Bio-Rad vertical system). The SDS-PAGE was performed at 10mA/gel for 30min, and then 45mA/gel, until the dye front reached the bottom of gels. After fixing in 50% ethanol, 12% acetate solution and staining with silver nitrate, gels were scanned at 300dpi resolution and the images were analysed with Image Master Platinum^™^ software (GE Health care).

Protein spots with *p* < 0.05 and > 1.3-fold change were considered significant. Spots of interest were picked from sliver-stained gels, followed by in-gel trypsin digestion and peptide extraction [35] for protein identification by tandem mass spectrometry analysis using ABI 4800 MALDI TOF/TOF^™^ MS Analyzer (Applied Biosystems, Foster City, CA, USA). The combined peptide mass fingerprinting (PMF) and MS/MS peptide fragmention data were submitted to the NCBInr database and SwissProt database using the software MASCOT version 2.2 (Matrix Science). For all significant protein identifications, both protein and total ion scores were above or equal to C.I. 99%.

### Western blot analyses

The striatum was homogenized at 4°C in lysis buffer (1:5,wt/vol) containing 1mM EDTA and 20mM phenylmethylsulphonyl fluoride. The proteins from the resultant supernatant were determined (Bio-Rad Protein Assay) for Sodium Dodecyle-Sulphate Polyacrylamide gel electrophoresis (SDS-PAGE). Protein (20ug/lane) was subjected to electrophoresis on a 10% (wt/vol) polyacrylamide gel in SDS, and gels subsequently processed for electroblotting to polyvinylidene difluoride (PVDF) membranes. The blotted PVDF membranes were saturated with 5% (wt/vol) of skimmed milk in Tris Buffer Saline, PH 7.4 and 0.1% (vol/vol) of Tween 20 for 1h at room temperature. The membranes were sequentially incubated with primary antibodies to anti-Calcineurin B (rabbit polyclonal IgG antibody, 1:500 dilution, Cat# ab136526, Abcam); anti-GAD 67 (rabbit polyclonal IgG antibody, 1:1000 dilution, Cat# ab52249, Abcam) and anti-GAD 65 (rabbit polyclonal IgG antibody, 1:500 dilution, Cat# ab75750, Abcam) overnight at 4°C following by a peroxidase-labelled anti-mouse/rabbit IgG (1:2000 dilution, Boehringer Mannheim, Germany) for 1h at room temperature. After thorough washing, positive bands were revealed using ECL western blotting detection reagents and autoradiography film (Amersham, Biosciences, UK). The intensities of the bands were quantified using IMAGE QUANT software (Molecular Dynamics, USA).

### Statistics

Behavioural results from each task were analysed using a GLM running in SPSS22 with ‘Dose’ of drug (saline, 10 μg/kg, 100 μg/kg, 1000 μg/kg oxytocin), ‘Sex’ and ‘Repeat exposure’ to oxytocin/saline (where appropriate) as between subject factors. Where there was a significant main effect of Sex, data were analysed in each Sex separately. If there was no effect of repeat exposure, data were combined for further analyses. Significant findings observed using GLM were explored using post-hoc Bonferroni tests were appropriate.

In PPI, drug treatment may alter the baseline startle response to pulse stimuli. This impacts upon subsequent analysis of %PPI [30]. Therefore, our PPI design included 3 different pulse conditions so that, should baseline startle reactivity to any pulse condition be significantly altered by oxytocin, that pulse condition could be excluded from further analysis of %PPI.

Multivariate analysis using partial least squares-discriminant analysis (PLS-DA, SIMCA 12.0, Umetrics, Umea, Sweden) was performed to examine whether C57BL/6N mice with oxytocin exposure could be discriminated from those with saline exposure on the basis of full proteome profile [36]. Subsequently Analysis of Variance running in the standard in multivariate data analysis (SIMCA-P+ 12.0, Umetrics) was used to identify proteins with significant up or down regulation in the oxytocin treated group compared to saline treated group. Protein expression differences in Western Blot were analysed using t-tests.

## Results

### Behaviour

In saline treated animals, the order of testing had no effect on any test.

#### Pulse-alone reactivity (Baseline startle reaction)

There was significant effect of sex on baseline pulse reactivity (F _(3, 100)_ = 28.883, *p* = 0.000); and a significant interaction of sex × dose (F _(3, 100)_ = 4.243, *p* = 0.007). However, previous oxytocin exposure had no impact on the baseline startle response (*p* = 0.214), therefore data from mice exposed to a single or repeated dose was combined for further analysis in each sex separately. This analysis indicated that male mice had higher startle responses than females at all doses of oxytocin (*p* < 0.01 for each dose).

**Fig 4:**
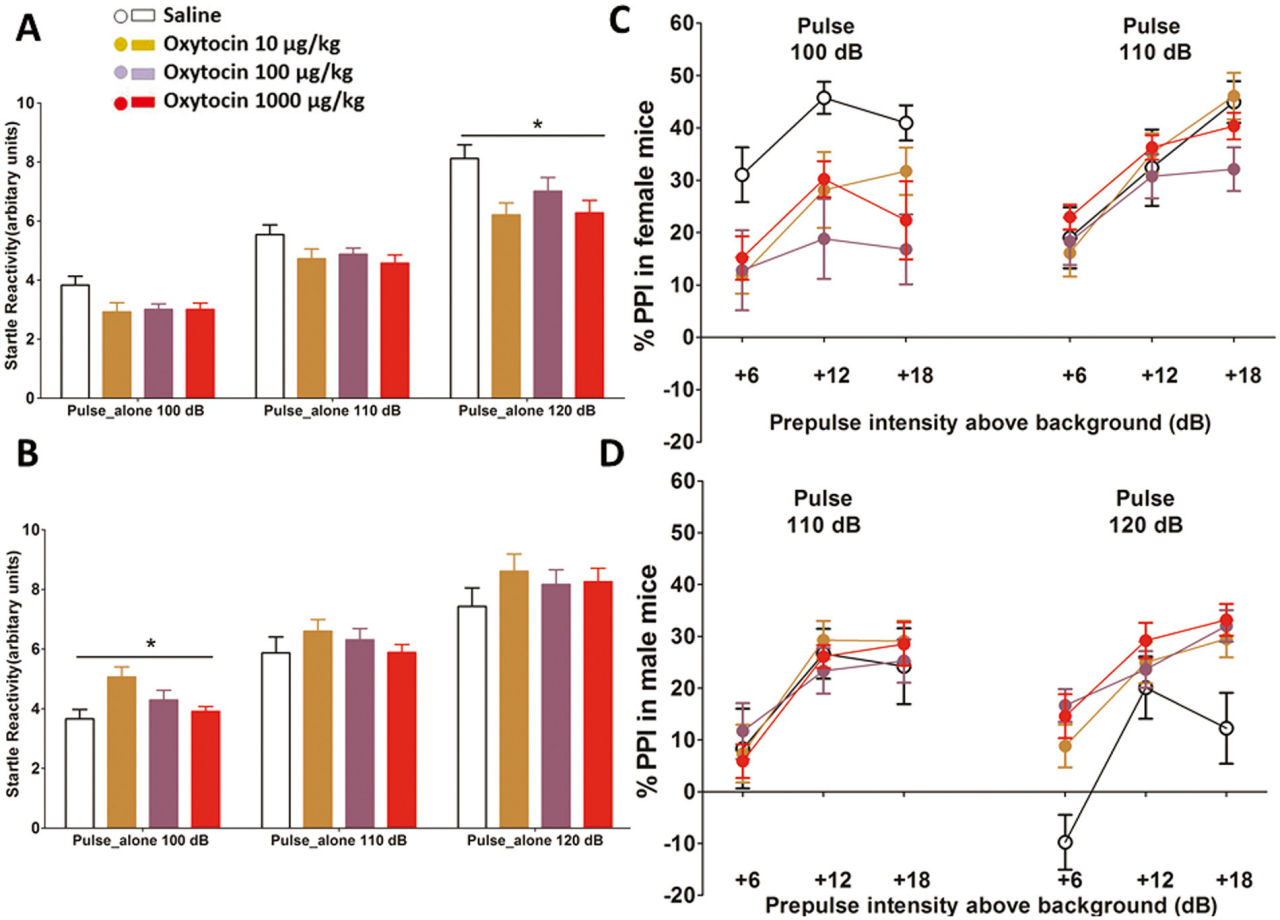
PPI test. (A) Oxytocin attenuated the baseline startle response to 120dB pulse in female mice. (B) Oxytocin increased the baseline startle response to 100dB pulse in male mice. (C) 100 μg/kg oxytocin attenuated PPI in females. (D) All doses of oxytocin improved PPI in males. Error bars refer to ± SEM.

Post-hoc analyses in each sex subsequently revealed that oxytocin altered startle responsivity at only one pulse condition in each sex; lowering the startle response at pulse 120 dB stimulus in females (F _(3, 54)_ = 3.330, *p* = 0.026, Fig 4A); increasing the startle response at pulse 100 dB in male mice (F _(3, 54)_ = 4.157, *p* = 0.010, Fig 4B). Therefore PPI analyses were conducted in each sex separately and data from pulse 120dB in females and pulse 100dB in males was excluded.

#### PPI

Previous oxytocin exposure had no impact on PPI (*p* = 0.957 in females; *p* = 0.393 in males), data from mice exposed to a single or repeated dose was combined for further analysis.

Oxytocin attenuated PPI in females (F _(3, 54)_ = 3.546, p = 0.020), especially following 100 μg/kg (Boneferroni test; p = 0.012, Fig 4C). In males, there was a significant dose x pulse interaction (F _(3, 54)_ = 3.120, p = 0.033); oxytocin improved PPI at pulse 120dB (F _(3, 54)_ = 5.112, *p* = 0.003), at all doses (post-hoc Bonferroni tests, p< 0.01 Fig 4D).

#### Novel object recognition test

Oxytocin increased time spent exploring objects at first presentation (F _(3, 108)_ = 4.292, *p* = 0.007), especially at doses of 100 μg/kg and 1000 μg/kg oxytocin (Bonferroni tests: *p* = 0.017 and *p* = 0.033 respectively). There was no effect of Sex on this measure.

**Fig 5:**
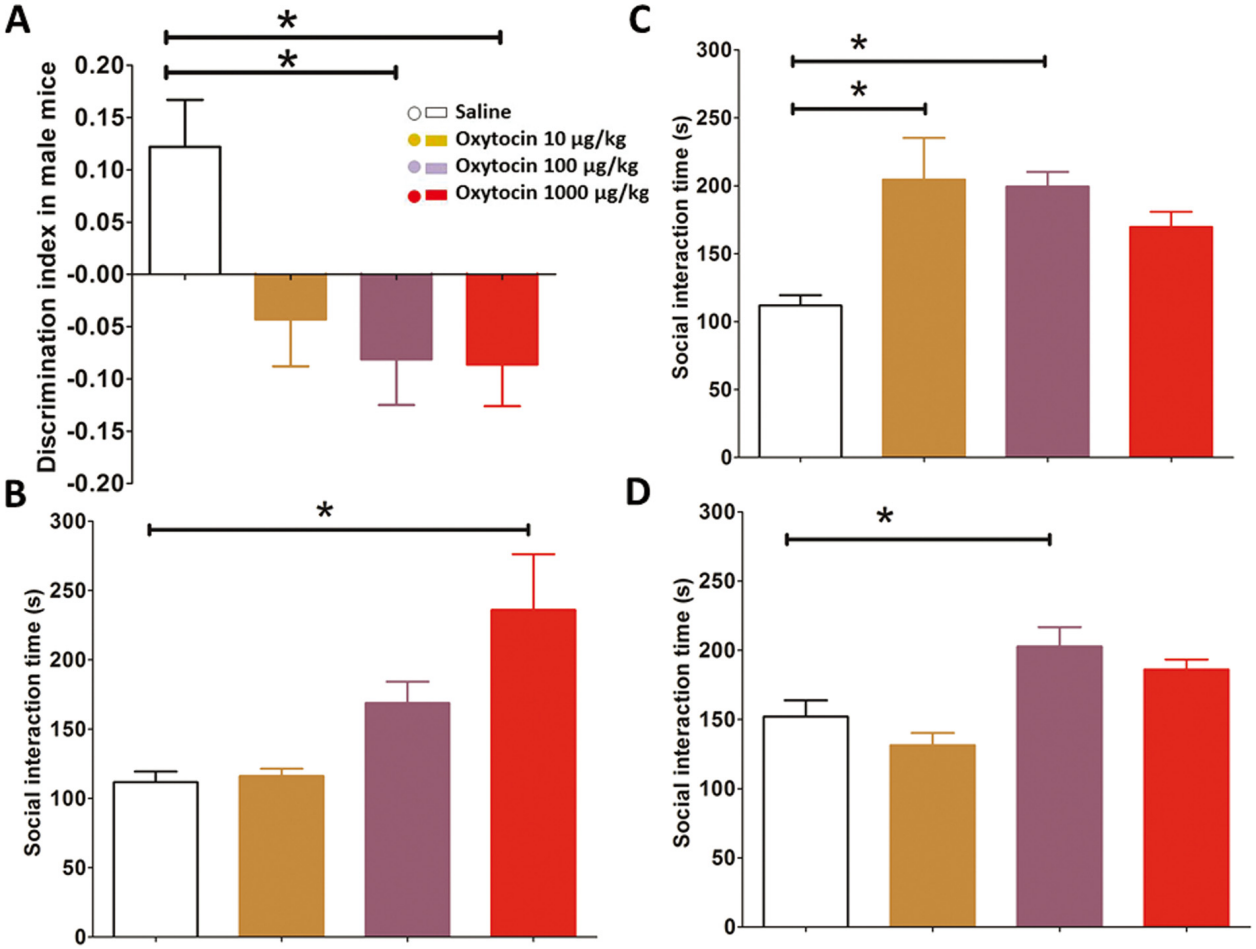
Novel object recognition and social interaction test. (A) 100 μg/kg and 1000 μg/kg oxytocin disrupted recognition memory in males. 1000 μg/kg single exposure (B) and 10 μg/kg and 100 μg/kg repeated exposure to oxytocin (C) increased social interaction in females. (D) 100 μg/kg oxytocin increased social interaction in males. Error bars refer to ± SEM. * post-hoc testing: *p<*0.05.

Analysis of the novelty discrimination index revealed a significant sex × dose interaction approached significance (F _(3, 108)_ = 2.628, *p* = 0.054). Post-hoc analyses in each sex separately revealed oxytocin did not alter this index in females but disrupted recognition memory in males (F _(3, 54)_ = 3.869, *p* = 0.014) at both 100 μg/kg and 1000 μg/kg doses (Bonferroni, (*p* = 0.023 and *p* = 0.019 respectively, Fig 5A).

#### Social interaction test

Oxytocin increased social interaction (F _(3, 100)_ = 10.914, *p* = 0.000) but there were significant interactions of dose with sex (F _(3, 100)_ = 2.827, *p* = 0.047); exposure (F _(3, 100)_ = 4.007, *p* = 0.009); and sex × exposure (F _(3, 100)_ = 3.365, *p* = 0.022) on this measure. Social interaction was therefore examined in each sex separately.

Oxytocin increased social interaction time in females (F _(3, 50)_ = 5.281, *p* = 0.003) and the dose × previous exposure interaction was significant (F _(3, 50)_ = 4.789, *p* = 0.005). Single doses of oxytocin increased social interaction time in females (F _(3, 25)_ = 5.942, *p* = 0.03). This result was driven by the 1000 μg/kg dose (post-hoc Bonferroni *p* = 0.012; Fig 5B). Previous exposure to oxytocin also increased social interaction time (F _(3, 25)_ = 3.635, *p* = 0.027), especially at doses of 10 μg/kg and 100 μg/kg oxytocin (Bonferroni tests: *p* = 0.034 and *p* = 0.049 respectively, Fig 5C).

Oxytocin increased social interaction time in male mice (F _(3, 50)_ = 9.779, *p* = 0.000) regardless of pre-exposure. Therefore data from groups exposed to a single or repeated dose of oxytocin were combined. Post-hoc Bonferroni test confirmed that 100 μg/kg oxytocin significantly increased social interaction time in male mice (*p* = 0.019, Fig 5D).

### Open field and amphetamine challenge test

***Basal locomotor activity*:** All mice had been given three doses of oxytocin or saline before the open field and amphetamine challenge (Fig 3); all had completed PPI, novel object and social interaction paradigms. The total distance moved in a one-hour open field test provided a measure of basal exploratory locomotor activity. Following an injection of oxytocin or saline, locomotion was measured for 30 minutes. Finally, each animal received 2.5 mg/kg amphetamine and locomotion measured for 60 minutes.

**Fig 6:**
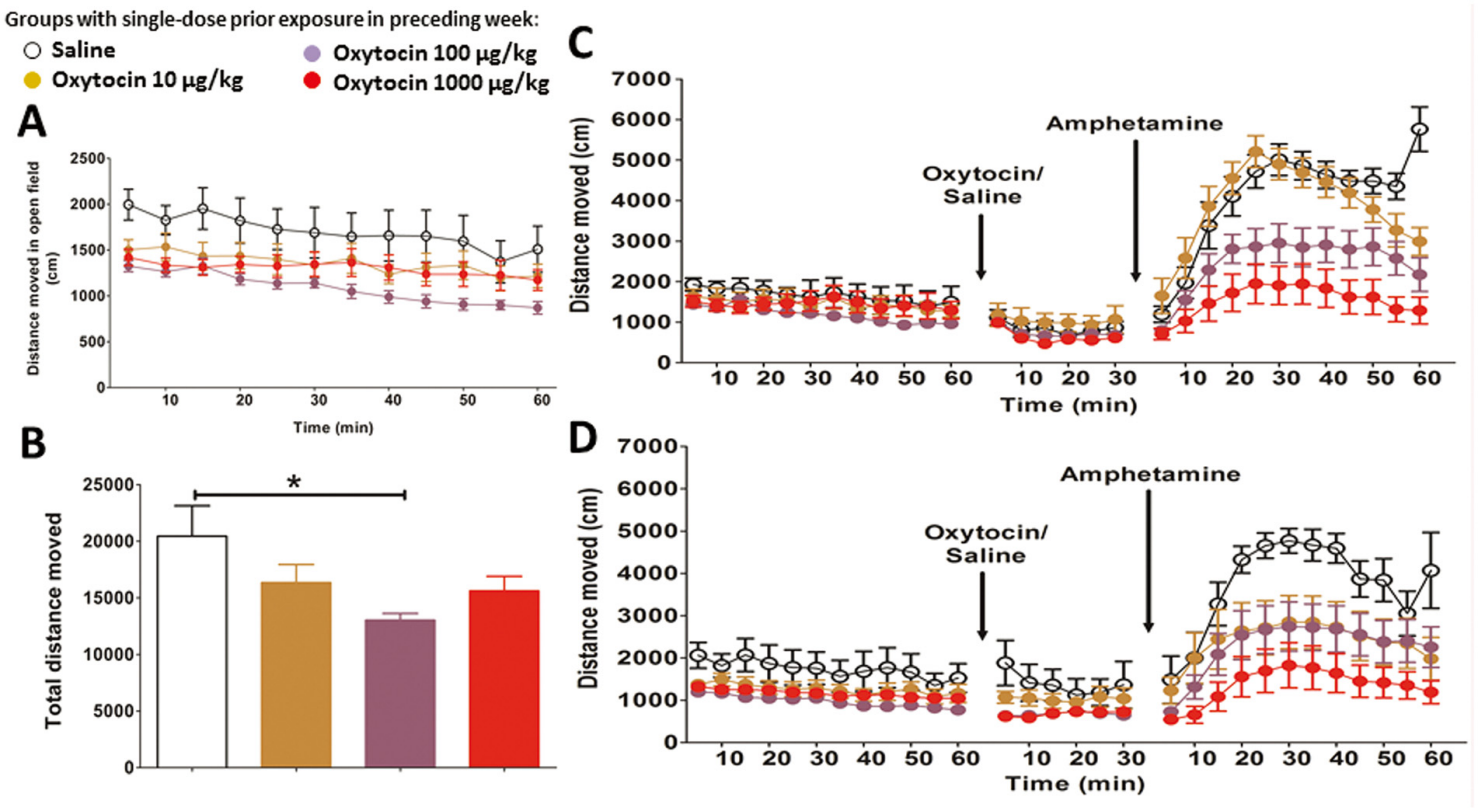
Open field and amphetamine test. (A) Distances moved in each 5-min time bin at baseline. (B) Total distance moved in 1 hour at baseline. Previous exposure to oxytocin lowered baseline activity in both sexes. (C) 100 μg/kg and 1000 μg/kg doses of oxytocin significantly suppressed amphetamine activity in females. (D) 1000μg/kg Oxytocin exposure significantly suppressed amphetamine activity in males. Error bars refer to ± SEM. *post-hoc testing: *p<*0.05.

Previous exposure to oxytocin lowered baseline activity in both sexes (F _(3, 100)_ = 3.649, *p* = 0.015), especially at 100 μg/kg (*p* = 0.009). There was no effect of an additional dose of oxytocin before testing (Fig 6A and 6B).

***Amphetamine-induced locomotor activity*:** Oxytocin significantly attenuated the response to amphetamine (F _(3, 100)_ = 11.743, *p* < = 0.001). There was no effect of an additional dose of oxytocin before testing. As previous oxytocin exposure altered baseline activity, the data were reanalysed with baseline distance entered as a covariate. This did not alter the pattern of results; thus differences in baseline activity could not explain attenuation of amphetamine-induced hyperlocomotion by oxytocin. There was however a significant time × dose × sex interaction in the amphetamine response (F _(33, 1167)_ = 2.115, *p* = 0.000), therefore, post-hoc analysis examined each sex separately.

Oxytocin attenuated the response to amphetamine in females (F _(3, 54)_ = 10.477, *p* = 0.000), especially at 100 μg/kg (*p* = 0.033) and 1000 μg/kg doses (*p* = 0.000, Fig 6C). It also attenuated the response to amphetamine in males (F _(3, 54)_ = 3.846, *p* = 0.015), particularly at the highest dose (1000μg/kg) (*p* = 0.008, Fig 6D). As 3 repeated weekly doses of 1000ug/kg oxytocin caused a clear suppression of amphetamine-induced locomotion in both male and female mice, this dose regime was chosen for the 2D proteomics analysis.

### Striatum proteomics

**Fig 7:**
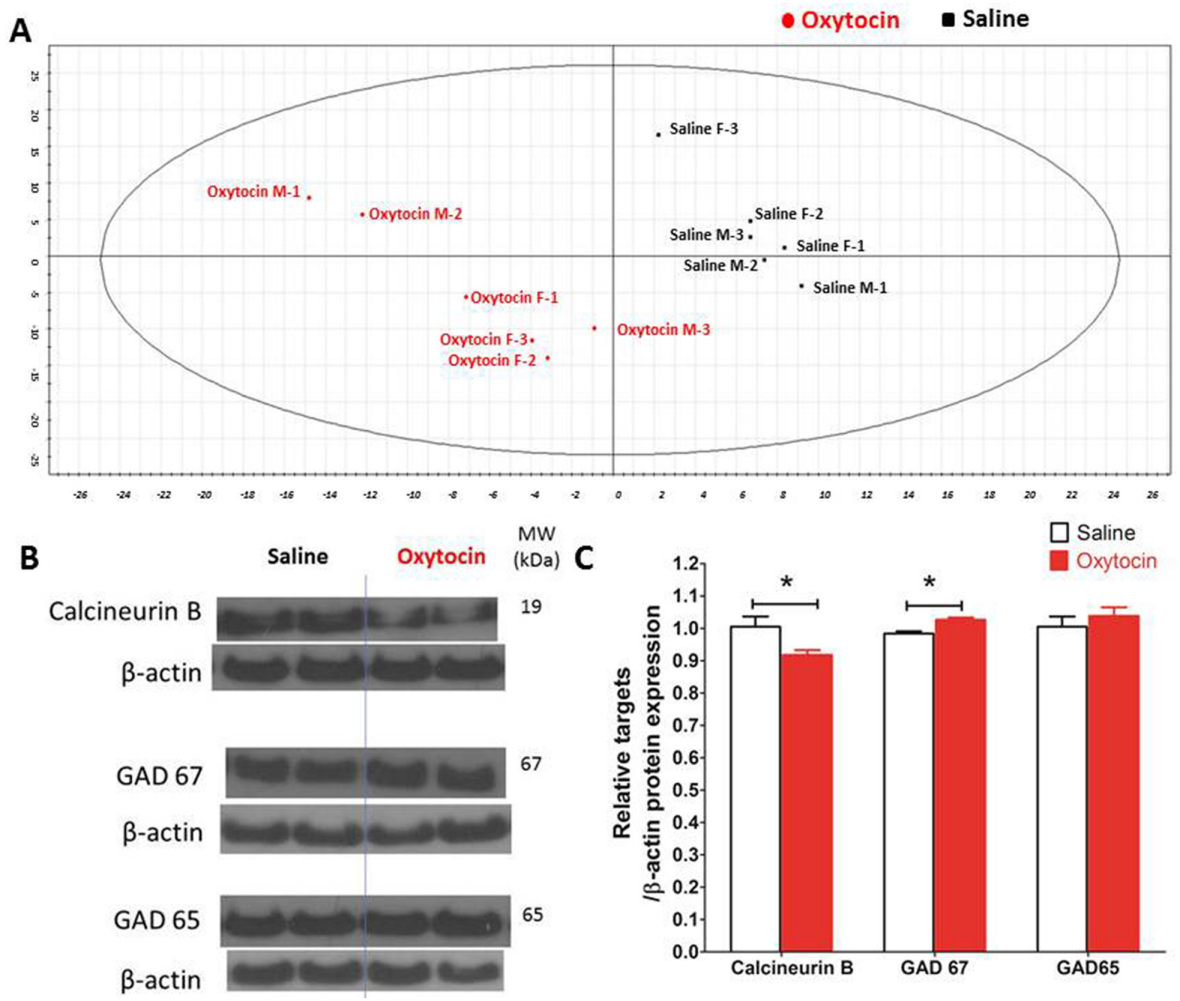
Striatum proteomics and western blot studies. (A) Multivariate analysis: partial least squares—discriminative analysis (PLS-DA) carried out using SIMCA-P+12.0 software (Umetrics). The striatal protein profile following oxytocin exposure could be clearly differentiated from saline (controls). The XY axis shows the coordinates of the protein distribution in the partial least squares—discriminative analysis (B) Western blot gel images of target proteins. (C) The relative expression of the target proteins/β-actin, Columns show mean±SEM (*n* = 9 for saline exposure; *n* = 8 for oxytocin exposure). * *p*<0.05.

A total of 811 individual protein spots were visualized across 12 gels (saline: *n* = 3 males, *n* = 3 females; oxytocin: *n* = 3 males, *n* = 3 females). All spots were included in a partial least squares-discriminant analysis (PLS-DA), which clearly separated protein expression in mice exposed to oxytocin from saline controls (Fig 7A).

Analysis of covariance confirmed significant drug-related differences in the expression of 15 proteins (Table 1). Western blot analysis of striatal tissue from animals used for behavioural testing confirmed that a protein identified in 2D proteomics, calcineurin, was significantly lowered by oxytocin.

**Table 1:** Proteins with differences in expression levels following 3 injections of oxytocin 1000 μg/kg or saline, at an interval of 1 week apart.

Spot No.	Fold difference	*p* value	Protein name	NCBI no.	Previous reported change with antipsychotics
33	1.6651	0.0178	protein DJ-1	gi|55741460	Up [74]
34	1.7887	0.0132	ubiquitin carboxyl-terminal hydrolase isozyme L3	gi|139948802	(No report)
37	1.3999	0.0453	alpha-enolase	gi|34784434	Up [75]
42	1.9003	0.0067	D-3-phosphoglycerate dehydrogenase	gi|986918	Up [76]
51	1.6083	0.0433	septin-6 isoform 1	gi|293597553	Up [76]
80	1.8686	0.0174	gamma-enolse	gi|7305027	Up [76]
81	2.1161	0.0329	Rho GDP-dissociation inhibitor 1	gi|31982030	Up [77]
95	1.8751	0.0372	14-3-3 protein zeta/delta isoform 1	gi|6756041	Up [77]
100	1.9886	0.0290	dihydrolipoyllysine-residue succinyltransferase component of 2-oxoglutarate dehydrogenase complex, mitochondrial	gi|21313536	(No report)
109	2.0968	0.0490	triosephosphate isomerase, partial	gi|1864018	Down [78]
127	2.2133	0.0049	dihydropyrimidinase-related protein 2	gi|40254595	Up [75]
145	1.9783	0.0026	tubulin alpha-1C chain	gi|6678469	Up [76]
275	1.9778	0.0273	ATP synthase, H+ transporting, mitochondrial F1 complex, alpha subunit, isoform 1	gi|148677501	Up [74]
322	-6.1599	0.0031	ornithine aminotransferase, mitochondrial precursor	gi|8393866	(No report)
330	-2.5450	0.0275	calcineurin subunit-B	gi|4506025	Down [78]

Fold differences in protein expression in the oxytocin group relative to the saline group are shown in column 2. Column 6 (final column) indicates how changes in protein expression elicited by oxytocin compare to those reposted to follow antipsychotic treatment in post-mortem studies of schizophrenia and/or rat models (final column).

2D-gel results were confirmed by Western blot analysis in a selected target protein, calcineurin. In addition, because 2D-gel analysis suggested that oxytocin may act upon GABA-glutamate pathways, we examined the effects of oxytocin on glutamic acid decarboxylase (GAD65 and GAD67) post-hoc. GAD catalyses the conversion of glutamate to GABA, and is a marker for GABAergic neurons. Both isoforms have been reported to be lower in schizophrenia and related neurodevelopmental disorders [37] and dopamine inhibits striatal GAD67 [38]. We expected that an ‘anti-psychotic’ effect of oxytocin would include up-regulation of GAD67 in striatum of C57BL/6N mice.

In addition, GAD67 expression (but not GAD65) was significantly elevated by oxytocin (Fig 7B and 7C).

## Discussion

Oxytocin has been proposed to be a potential treatment for neurodevelopmental conditions. In this study, peripherally administered oxytocin improved PPI and social interaction in males and attenuated the response to amphetamine in open field test in both sexes. This adds to an increasing body of evidence that oxytocin promotes pro-social behaviour and also modulates non-social behaviour [2].

There were also global differences (PLS-DA analysis) in protein expression elicited by a 1000 μg/kg dose of oxytocin compared to saline. Oxytocin generally, had effects broadly similar to conventional anti-psychotics (Table 1). The proteins altered by oxytocin could be categorized as those modulating glutamatergic, GABAergic or dopaminergic signalling systems, and cytoskeleton components. Such expression changes may in part explain the behavioural differences observed following oxytocin administration.

### PPI

Deficits in sensorimotor gating measured using PPI are associated with neurodevelopmental disorders. We selected the C57BL/6N mouse strain for this study because it has lower PPI than many other in-bred strains [39] and has been suggested to model aspects of schizophrenia [40]. Thus, any improvement elicited by oxytocin should be evident above the low baseline PPI expected in these animals.

Oxytocin increased PPI in male mice—a direction of effect similar to that reported using conventional anti-psychotics [41]. This result is partly consistent with previous reports that high doses of oxytocin (1000μg/kg) improve PPI in male Brown-Norway rats [42] and reverse PPI deficits induced by NMDA antagonists in rats [28]. However, the sensitivity of rodents to oxytocin is likely to be species and strain specific [43], and to be dependent upon the parameters of the PPI paradigm [44].

Although oxytocin attenuated PPI in female mice, as can be seen from Fig 4C and 4D, the levels of PPI in female mice were higher than even males treated with oxytocin. Thus oxytocin improved a behaviour ‘impaired’ in males, but had limited impact upon behaviour essentially intact in females. The latter observation fits with clinical evidence that PPI is intact in females with schizophrenia [45].

Our results contrast with the previous observation that neither oxytocin knockout [3] nor oxytocin receptor knockout [46] disrupts PPI. Thus, oxytocin is not ‘necessary’ for PPI, but does alter it. One mechanism for this action may be through the glutamate-GABA system which contributes to PPI [47] and is thought to be modulated by oxytocin [48]. Consistent with this, we found that oxytocin down-regulated ornithine aminotransferase, which catalyses the transamination of ornithine to glutamate [49]; and, up-regulated 14-3-3 protein zeta/delta (or KCIP-1), involved in GABA_B_ receptor coupling and dopamine D2 receptor inhibition [50]. In addition, oxytocin increased the level of GAD67 which reflects intraneuronal gamma-aminobutyric acid levels [51]. Thus, we speculate that the net effect of oxytocin may be to promote GABAergic signalling and restrain glutamate and dopamine activity, improving PPI.

### Novel object recognition

In the first step of the novel object paradigm, oxytocin increased time spent exploring the objects, possibly representing an anxiolytic action [52]. However, the novel object component of this paradigm indexes memory for a familiar object and oxytocin disrupted recognition memory in males (Fig 3A) without altering this measure in females.

Again a pro-GABA action may contribute through calcineurin and KCIP-1 regulation of GABA_A_ and GABA_B_ receptors respectively. In line with this, GABA_A_ [53] and GABA_B_ [54] agonists impair recognition memory and calcineurin knockout has also been reported to impair memory in mice [55].

### Social interaction

Oxytocin increased social interaction time in both females and males, but the dose response was sex-dependent (Fig 5B–5D). Oxytocin had a cumulative effect on social interaction in female mice; whereas previous exposure to oxytocin in males was no different to a single exposure. These facilitatory effects of oxytocin on social interaction are consistent with studies in oxytocin knock-out mice [56]. As discussed above, it is possible that oxytocin triggered changes in GABA signalling contribute. For example, GABA_A_ agonists increase social interaction time in rats, and antagonists do the opposite [57]. No dose of oxytocin used reduced social interaction, ruling out sedative effects.

### Open field activity and response to amphetamine

Amphetamine is an indirect-acting dopamine agonist that increases synaptic dopamine; patients with schizophrenia are particularly vulnerable to amphetamine. Here, oxytocin suppressed amphetamine-induced locomotion in both sexes (even controlling for baseline activity differences). A possible explanation is that oxytocin promotes GABA-medicated inhibition of striatal dopamine release [58], and the increased level of GAD67 following oxytocin exposure points to increased GABAergic inhibitory activity. However, our proteomics results suggest additional pathways for oxytocin to limit dopamine mechanisms. For example, the 14-3-3 protein zeta/delta, up-regulated by oxytocin, is known to inhibit PKC [59] and PKC inhibitors have been shown to block amphetamine-induced dopamine release from rat striatal synaptoneurosome [60].

The attenuation of the amphetamine response occurred in mice previously exposed to oxytocin, with or without an additional acute dose of oxytocin (Fig 6). This indication that intermittent exposure to oxytocin can have ‘lasting’ effects will be important to explore further in light of recent evidence that chronic administration of oxytocin may have undesirable effects, disrupting social bonding in male voles [61].

### Effects of oxytocin on other proteins

In addition to actions on the glutamate-GABA systems, oxytocin had other wide-ranging effects including on proteins involved in cell structure and plasticity—tubulin-α dihydropyrimidinase-related protein 2 (DRP-2) and D-3-phosphoglycerate dehydrogenase (3-PGDH) [62–64]; and mitochondrial function—upregulating ATP synthase subunit alpha [65]; DJ-1 [66], and enolase (linked to recovery of cerebral metabolism in patients with schizophrenia treated with anti-psychotic medication [67]).

### Limitations

There are some limitations to our current study. To reduce the number of animals used, we tested each mouse in every behavioural paradigm design. The order of testing did not impact upon findings in the animals exposed to saline, and previous exposure to oxytocin did not influence results of PPI in either sex or social interaction in males. However, previous exposure was sufficient to suppress the amphetamine response in both sexes and improve social interaction in females. Unfortunately, our study was not designed to test the effect of an acute exposure to oxytocin in naïve animals in the amphetamine challenge (without greatly increasing the number of animals used).

The half-life of oxytocin is 10-15min [68], but only about 0.1% of oxytocin can cross the blood brain barrier [69]. Therefore the effects studied here are most liekly indirect. The timing of oxytocin administration was challenging to match across tasks. For example, in PPI, as in other drug studies using this paradigm, oxytocin was administered 10 mins before the task, but data collected in the task for a further 45 mins. In the social interaction and amphetamine challenge tests the effects of oxytocin were captured after 30mins. Thus for these tasks the effects of oxytocin from 30mins would have been captured. However, oxytocin was given after the ‘learning phase’ of the object recognition task to establish if it had any effect on the consolidation stage.

We observed subtle sex differences in the oxytocin dose response. However, the study was not primarily designed to compare sex differences in response to oxytocin. Rather the study was designed to look at the effects of oxytocin and both male and female animals were included. This is increasingly recognised as important and the NIH now requires the reporting of plans for a balance of male and female animals in preclinical studies unless single sex inclusion is specifically warranted [70].

Unfortunately, the underlying differences in baseline behaviours make interpretation difficult. For example, as previously reported, female C57BL/6N mice had higher levels of PPI than males [71]. This is not thought to be a consequence of the oestrous cycle which does not alter PPI and anxiety-related behaviours in female C57BL mice [72]. Male C57BL/6N mice also had longer social interaction times than female mice (Fig 5B, 5C&5D); possibly meaning they were less anxious than females. However, in addition to these baseline behavioural differences between the sexes, the differential response to oxytocin may also be a consequence of sex differences in expression of oxytocin and its receptors [7] and further work is therefore needed to clarify sex effects of oxytocin.

We only examined 2D protein differences in the striatum in response to repeated exposure to 1000 μg/kg oxytocin in naïve animals, and we cannot directly link differences to phenotypic differences. However, confirmatory Western blot studies carried out using tissue from animals involved in the behavioural experiments adds weight to the likelihood that the proteins implicated contribute to the behavioural differences observed. Protein expression differs across brain regions [73] and we do not know if our results generalize across the brain. Nor did we examine protein expression in each sex separately. However, the PLS-DA suggests the distinct protein profile in animals exposed to oxytocin and saline involved both sexes and the effects of oxytocin on social interaction and amphetamine challenge tasks were broadly similar in both sexes. Lastly, the proteomics approach used here was a standard analysis that does not fully sample intracellular organelles. The future separation of subcellular fractions would be useful.

## Conclusion

Oxytocin significantly improves social and non-social behaviours in an experimental animal setting. It elicits a pattern of changes in striatal protein expression similar to that triggered by conventional antipsychotics. We hope that this study encourages further research into the clinical application of this peptide hormone, which may extend treatment options across a spectrum of neurodevelopmental conditions.

## References

[1] Gimpl G, Fahrenholz F. The oxytocin receptor system: structure, function, and regulation. Physiological reviews. 2001;81(2):629–83. 11274341 10.1152/physrev.2001.81.2.629

[2] Lee H-J, Macbeth AH, Pagani JH. Oxytocin: the great facilitator of life. Progress in neurobiology. 2009;88(2):127–51. 10.1016/j.pneurobio.2009.04.001 19482229 PMC2689929

[3] Caldwell H, Stephens S, Young rW. Oxytocin as a natural antipsychotic: a study using oxytocin knockout mice. Molecular psychiatry. 2008;14(2):190–6. 10.1038/sj.mp.4002150 18227836

[4] Feifel D, Macdonald K, Nguyen A, Cobb P, Warlan H, Galangue B, et al Adjunctive intranasal oxytocin reduces symptoms in schizophrenia patients. Biological psychiatry. 2010;68(7):678–80. 10.1016/j.biopsych.2010.04.039 20615494

[5] Tomizawa K, Iga N, Lu Y-F, Moriwaki A, Matsushita M, Li S-T, et al Oxytocin improves long-lasting spatial memory during motherhood through MAP kinase cascade. Nature neuroscience. 2003;6(4):384–90. 12598900 10.1038/nn1023

[6] Lee PR, Brady DL, Shapiro RA, Dorsa DM, Koenig JI. Social interaction deficits caused by chronic phencyclidine administration are reversed by oxytocin. Neuropsychopharmacology. 2005;30(10):1883–94. 15798779 10.1038/sj.npp.1300722

[7] Carter CS. Sex differences in oxytocin and vasopressin: implications for autism spectrum disorders? Behavioural brain research. 2007;176(1):170–86. 17000015 10.1016/j.bbr.2006.08.025

[8] Loth E, Poline J-B, Thyreau B, Jia T, Tao C, Lourdusamy A, et al Oxytocin receptor genotype modulates ventral striatal activity to social cues and response to stressful life events. Biological psychiatry. 2013. 10.1016/j.biopsych.2013.07.043 24120094

[9] Langen M, Schnack HG, Nederveen H, Bos D, Lahuis BE, de Jonge MV, et al Changes in the developmental trajectories of striatum in autism. Biological psychiatry. 2009;66(4):327–33. 10.1016/j.biopsych.2009.03.017 19423078

[10] Tricomi E, Rangel A, Camerer CF, O’Doherty JP. Neural evidence for inequality-averse social preferences. Nature. 2010;463(7284):1089–91. 10.1038/nature08785 20182511

[11] Leung M, Cheung C, Yu K, Yip B, Sham P, Li Q, et al Gray matter in first-episode schizophrenia before and after antipsychotic drug treatment. Anatomical likelihood estimation meta-analyses with sample size weighting. Schizophrenia Bulletin. 2011;37(1):199–211. 10.1093/schbul/sbp099 19759093 PMC3004197

[12] Yu K, Cheung C, Leung M, Li Q, Chua S, McAlonan G. Are bipolar disorder and schizophrenia neuroanatomically distinct? An anatomical likelihood meta-analysis. Frontiers in human neuroscience. 2010;4. 21103008 10.3389/fnhum.2010.00189 PMC2987512

[13] Cheung C, Yu K, Fung G, Leung M, Wong C, Li Q, et al Autistic disorders and schizophrenia: related or remote? An anatomical likelihood estimation. PloS one. 2010;5(8):e12233 10.1371/journal.pone.0012233 20805880 PMC2923607

[14] Kevin KY, Cheung C, Chua SE, McAlonan GM. Can Asperger syndrome be distinguished from autism? An anatomic likelihood meta-analysis of MRI studies. Journal of psychiatry & neuroscience: JPN. 2011;36(6):412. 21406158 10.1503/jpn.100138 PMC3201995

[15] Swerdlow N, Geyer M, Braff D. Neural circuit regulation of prepulse inhibition of startle in the rat: current knowledge and future challenges. Psychopharmacology. 2001;156(2–3):194–215. 11549223 10.1007/s002130100799

[16] Peeke HV, Herz MJ. Caudate nucleus stimulation retroactively impairs complex maze learning in the rat. Science. 1971;173(3991):80–2. 4932263 10.1126/science.173.3991.80

[17] Wyers EJ, Peeke HV, Williston JS, Herz MJ. Retroactive impairment of passive avoidance learning by stimulation of the caudate nucleus. Experimental Neurology. 1968;22(3):350–66. 4884398 10.1016/0014-4886(68)90002-2

[18] Brenes JC, Rodríguez O, Fornaguera J. Differential effect of environment enrichment and social isolation on depressive-like behavior, spontaneous activity and serotonin and norepinephrine concentration in prefrontal cortex and ventral striatum. Pharmacology Biochemistry and Behavior. 2008;89(1):85–93. 10.1016/j.pbb.2007.11.004 18096212

[19] King-Casas B, Tomlin D, Anen C, Camerer CF, Quartz SR, Montague PR. Getting to know you: reputation and trust in a two-person economic exchange. Science. 2005;308(5718):78–83. 15802598 10.1126/science.1108062

[20] Castner SA, Becker JB. Sex differences in the effect of amphetamine on immediate early gene expression in the rat dorsal striatum. Brain research. 1996;712(2):245–57. 8814899 10.1016/0006-8993(95)01429-2

[21] Castner SA, Xiao L, Becker JB. Sex differences in striatal dopamine: in vivo microdialysis and behavioral studies. Brain research. 1993;610(1):127–34. 8518920 10.1016/0006-8993(93)91225-h

[22] Konradi C, Heckers S. Antipsychotic drugs and neuroplasticity: insights into the treatment and neurobiology of schizophrenia. Biological psychiatry. 2001;50(10):729–42. 11720691 10.1016/s0006-3223(01)01267-7 PMC4205586

[23] Pakkenberg H, Fog R, Nilakantan B. The long-term effect of perphenazine enanthate on the rat brain. Some metabolic and anatomical observations. Psychopharmacologia. 1973;29(4):329–36. 4707024 10.1007/BF00429280

[24] Nielsen EB, Lyon M. Evidence for cell loss in corpus striatum after long-term treatment with a neuroleptic drug (flupenthixol) in rats. Psychopharmacology. 1978;59(1):85–9. 100820 10.1007/BF00428036

[25] Takeichi M. Electron Microscopic Morphometric Studies on Synaptic Vesicles of Long-Term CPZ-Administered Rat Striatum. Psychiatry and Clinical Neurosciences. 1985;39(2):185–92. 10.1111/j.1440-1819.1985.tb02902.x 4065762

[26] Bai O, Chlan-Fourney J, Bowen R, Keegan D, Li XM. Expression of brain-derived neurotrophic factor mRNA in rat hippocampus after treatment with antipsychotic drugs. Journal of neuroscience research. 2003;71(1):127–31. 12478621 10.1002/jnr.10440

[27] Deng MY, McAlonan GM, Cheung C, Chiu CP, Law CW, Cheung V, et al A naturalistic study of grey matter volume increase after early treatment in anti-psychotic naive, newly diagnosed schizophrenia. Psychopharmacology. 2009;206(3):437–46. 10.1007/s00213-009-1619-z 19641900

[28] Feifel D, Reza T. Oxytocin modulates psychotomimetic-induced deficits in sensorimotor gating. Psychopharmacology. 1999;141(1):93–8. 9952070 10.1007/s002130050811

[29] Li Q, Cheung C, Wei R, Hui ES, Feldon J, Meyer U, et al Prenatal immune challenge is an environmental risk factor for brain and behavior change relevant to schizophrenia: evidence from MRI in a mouse model. PloS one. 2009;4(7):e6354 10.1371/journal.pone.0006354 19629183 PMC2710518

[30] Csomor PA, Yee BK, Vollenweider FX, Feldon J, Nicolet T, Quednow BB. On the influence of baseline startle reactivity on the indexation of prepulse inhibition. Behavioral neuroscience. 2008;122(4):885 10.1037/0735-7044.122.4.885 18729642

[31] Vuillermot S, Weber L, Feldon J, Meyer U. A longitudinal examination of the neurodevelopmental impact of prenatal immune activation in mice reveals primary defects in dopaminergic development relevant to schizophrenia. The Journal of Neuroscience. 2010;30(4):1270–87. 10.1523/JNEUROSCI.5408-09.2010 20107055 PMC6633802

[32] Bevins RA, Besheer J. Object recognition in rats and mice: a one-trial non-matching-to-sample learning task to study'recognition memory'. Nature protocols. 2006;1(3):1306–11. 17406415 10.1038/nprot.2006.205

[33] Spruijt BM, Josephy M, Van Rijzingen I, Maaswinkel H. The ACTH (4–9) analog Org2766 modulates the behavioral changes induced by NMDA and the NMDA receptor antagonist AP5. The Journal of neuroscience. 1994;14(5):3225–30. 7910205 10.1523/JNEUROSCI.14-05-03225.1994 PMC6577486

[34] Meyer U, Nyffeler M, Yee BK, Knuesel I, Feldon J. Adult brain and behavioral pathological markers of prenatal immune challenge during early/middle and late fetal development in mice. Brain, behavior, and immunity. 2008;22(4):469–86. 18023140 10.1016/j.bbi.2007.09.012

[35] Wang Y, Lam KS, Lam JB, Lam MC, Leung PT, Zhou M, et al Overexpression of angiopoietin-like protein 4 alters mitochondria activities and modulates methionine metabolic cycle in the liver tissues of db/db diabetic mice. Molecular Endocrinology. 2007;21(4):972–86. 17213385 10.1210/me.2006-0249

[36] Karp NA, Griffin JL, Lilley KS. Application of partial least squares discriminant analysis to two-dimensional difference gel studies in expression proteomics. Proteomics. 2005;5(1):81–90. 15744836 10.1002/pmic.200400881

[37] Guidotti A, Auta J, Davis JM, Gerevini VD, Dwivedi Y, Grayson DR, et al Decrease in reelin and glutamic acid decarboxylase67 (GAD67) expression in schizophrenia and bipolar disorder: a postmortem brain study. Archives of general psychiatry. 2000;57(11):1061–9. 11074872 10.1001/archpsyc.57.11.1061

[38] Caboche J, Vernier P, Julien JF, Rogard M, Mallet J, Besson MJ. Parallel decrease of glutamic acid decarboxylase and preproenkephalin mRNA in the rat striatum following chronic treatment with a dopaminergic D1 antagonist and D2 agonist. Journal of neurochemistry. 1991;56(2):428–35. 1824860 10.1111/j.1471-4159.1991.tb08168.x

[39] Crawley J, Belknap JK, Collins A, Crabbe JC, Frankel W, Henderson N, et al Behavioral phenotypes of inbred mouse strains: implications and recommendations for molecular studies. Psychopharmacology. 1997;132(2):107–24. 9266608 10.1007/s002130050327

[40] Matsuo N, Takao K, Nakanishi K, Yamasaki N, Tanda K, Miyakawa T. Behavioral profiles of three C57BL/6 substrains. Frontiers in behavioral neuroscience. 2010;4. 20676234 10.3389/fnbeh.2010.00029 PMC2912075

[41] Vollenweider FX, Barro M, Csomor PA, Feldon J. Clozapine enhances prepulse inhibition in healthy humans with low but not with high prepulse inhibition levels. Biological psychiatry. 2006;60(6):597–603. 16997001 10.1016/j.biopsych.2006.03.058

[42] Feifel D, Shilling PD, Belcher AM. The effects of oxytocin and its analog, carbetocin, on genetic deficits in sensorimotor gating. European Neuropsychopharmacology. 2012;22(5):374–8. 10.1016/j.euroneuro.2011.09.004 21962914 PMC4208693

[43] Scott JP. Agonistic behavior of mice and rats: A review. American Zoologist. 1966;6(4):683–701. 4859807 10.1093/icb/6.4.683

[44] Braff D, Stone C, Callaway E, Geyer M, Glick I, Bali L. Prestimulus effects on human startle reflex in normals and schizophrenics. Psychophysiology. 1978;15(4):339–43. 693742 10.1111/j.1469-8986.1978.tb01390.x

[45] Kumari V, Aasen I, Sharma T. Sex differences in prepulse inhibition deficits in chronic schizophrenia. Schizophrenia research. 2004;69(2):219–35. 15469195 10.1016/j.schres.2003.09.010

[46] Swonger JM. Prepulse Inhibition of the Startle Reflex in Forebrain Oxytocin Receptor Knockout Mice: Kent State University; 2011.

[47] Takahashi K, Nagai T, Kamei H, Maeda K, Matsuya T, Arai S, et al Neural circuits containing pallidotegmental GABAergic neurons are involved in the prepulse inhibition of the startle reflex in mice. Biological psychiatry. 2007;62(2):148–57. 17027927 10.1016/j.biopsych.2006.06.035

[48] Jo Y-H, Stoeckel M-E, Freund-Mercier M-J, Schlichter R. Oxytocin modulates glutamatergic synaptic transmission between cultured neonatal spinal cord dorsal horn neurons. The Journal of neuroscience. 1998;18(7):2377–86. 9502799 10.1523/JNEUROSCI.18-07-02377.1998 PMC6793116

[49] Wroblewski JT, Blaker WD, Meek JL. Ornithine as a precursor of neurotransmitter glutamate: Effect of canaline on ornithine aminotransferase activity and glutamate content in the septum of rat brain. Brain research. 1985;329(1):161–8. 2858253 10.1016/0006-8993(85)90521-9

[50] Johnson SW, North RA. Two types of neurone in the rat ventral tegmental area and their synaptic inputs. The Journal of physiology. 1992;450(1):455–68. 1331427 10.1113/jphysiol.1992.sp019136 PMC1176131

[51] K R, DL M. The level of GAD 67 protein is highly sensitive to small increases in interpersonal gamma-rainout acid levels. J Neurochem. 1994;4;62(4):1375–81. 8133268 10.1046/j.1471-4159.1994.62041375.x

[52] Crawley JN. Exploratory behavior models of anxiety in mice. Neuroscience & Biobehavioral Reviews. 1985;9(1):37–44. 2858080 10.1016/0149-7634(85)90030-2

[53] Roberts E. GABA: the road to neurotransmitter status. Benzodiazepine/GABA receptors and chloride channels: structural and functional properties. 1986:1–39.

[54] Castellano C, Brioni JD, Nagahara AH, McGaugh JL. Post-training systemic and intra-amygdala administration of the GABA-B agonist baclofen impairs retention. Behavioral and neural biology. 1989;52(2):170–9. 2552976 10.1016/s0163-1047(89)90285-9

[55] Zeng H, Chattarji S, Barbarosie M, Rondi-Reig L, Philpot BD, Miyakawa T, et al Forebrain-specific calcineurin knockout selectively impairs bidirectional synaptic plasticity and working/episodic-like memory. Cell. 2001;107(5):617–29. 11733061 10.1016/s0092-8674(01)00585-2

[56] Ferguson JN, Aldag JM, Insel TR, Young LJ. Oxytocin in the medial amygdala is essential for social recognition in the mouse. The Journal of Neuroscience. 2001;21(20):8278–85. 11588199 10.1523/JNEUROSCI.21-20-08278.2001 PMC6763861

[57] Shekhar A, Katner J. Dorsomedial hypothalamic GABA regulates anxiety in the social interaction test. Pharmacology Biochemistry and Behavior. 1995;50(2):253–8. 10.1016/0091-3057(94)00307-5 7740065

[58] Scheel-Krüger J. Dopamine-GABA interactions: evidence that GABA transmits, modulates and mediates dopaminergic functions in the basal ganglia and the limbic system. Acta Neurologica Scandinavica. 1986. 3014800

[59] Wheeler-Jones C, Learmonth M, Martin H, Aitken A. Identification of 14-3-3 proteins in human platelets: effects of synthetic peptides on protein kinase C activation. Biochem J. 1996;315:41–7. 8670130 10.1042/bj3150041 PMC1217194

[60] Kantor L, Gnegy M. Protein kinase C inhibitors block amphetamine-mediated dopamine release in rat striatal slices. Journal of Pharmacology and Experimental Therapeutics. 1998;284(2):592–8. 9454802

[61] Bales KL, Perkeybile AM, Conley OG, Lee MH, Guoynes CD, Downing GM, et al Chronic intranasal oxytocin causes long-term impairments in partner preference formation in male prairie voles. Biological psychiatry. 2013;74(3):180–8. 10.1016/j.biopsych.2012.08.025 23079235 PMC3556198

[62] Wheal HV, Chen Y, Mitchell J, Schachner M, Maerz W, Wieland H, et al Molecular mechanisms that underlie structural and functional changes atthe postsynaptic membrane duringsynaptic plasticity. Progress in neurobiology. 1998;55(6):611–40. 9670221 10.1016/s0301-0082(98)00026-4

[63] Charrier E, Reibel S, Rogemond V, Aguera M, Thomasset N, Honnorat J. Collapsin response mediator proteins (CRMPs). Molecular neurobiology. 2003;28(1):51–63. 14514985 10.1385/MN:28:1:51

[64] Klomp LW, de Koning TJ, Malingré HE, van Beurden EA, Brink M, Opdam FL, et al Molecular characterization of 3-phosphoglycerate dehydrogenase deficiency—a neurometabolic disorder associated with reduced L-serine biosynthesis. The American Journal of Human Genetics. 2000;67(6):1389–99. 11055895 10.1086/316886 PMC1287916

[65] Fillingame RH. Molecular mechanics of ATP synthesis by F1F0-type H+-transporting ATP synthases. The bacteria. 1990;12:345–91.

[66] Taira T, Saito Y, Niki T, Iguchi-Ariga SM, Takahashi K, Ariga H. DJ-1 has a role in antioxidative stress to prevent cell death. EMBO reports. 2004;5(2):213–8. 14749723 10.1038/sj.embor.7400074 PMC1298985

[67] Szechtman H, Nahmias C, Garnett ES, Firnau G, Brown GM, Kaplan RD, et al Effect of neuroleptics on altered cerebral glucose metabolism in schizophrenia. Archives of General Psychiatry. 1988;45(6):523–32. 2897836 10.1001/archpsyc.1988.01800300019002

[68] Dawood M. Neurohypophyseal hormones Endocrinology of Pregnancy, 3rd Edition Philadelphia: Harper & Row 1983:204–28.

[69] Jones P, Robinson I. Differential clearance of neurophysin and neurohypophysial peptides from the cerebrospinal fluid in conscious guinea pigs. Neuroendocrinology. 1982;34(4):297–302. 7070597 10.1159/000123316

[70] Clayton JA, Collins FS. NIH to balance sex in cell and animal studies. Nature. 2014;509(7500):282–3. 24834516 10.1038/509282a PMC5101948

[71] Zhang X, Li Q, Wong N, Zhang M, Wang W, Bu B, et al Behaviour and prefrontal protein differences in C57BL/6N and 129 X1/SvJ mice. Brain research bulletin. 2015. 10.1016/j.brainresbull.2015.05.003 26003851

[72] Meziane H, Ouagazzal AM, Aubert L, Wietrzych M, Krezel W. Estrous cycle effects on behavior of C57BL/6J and BALB/cByJ female mice: implications for phenotyping strategies. Genes, Brain and Behavior. 2007;6(2):192–200. 10.1111/j.1601-183X.2006.00249.x 16827921

[73] Takao K, Kobayashi K, Hagihara H, Ohira K, Shoji H, Hattori S, et al Deficiency of Schnurri-2, an MHC enhancer binding protein, induces mild chronic inflammation in the brain and confers molecular, neuronal, and behavioral phenotypes related to schizophrenia. Neuropsychopharmacology. 2013;38(8):1409–25. 10.1038/npp.2013.38 23389689 PMC3682135

[74] Clark D, Dedova I, Cordwell S, Matsumoto I. Altered proteins of the anterior cingulate cortex white matter proteome in schizophrenia. PROTEOMICS-Clinical Applications. 2007;1(2):157–66. 10.1002/prca.200600541 21136665

[75] Pennington K, Föcking M, McManus C, Pariante C, Dunn M, Cotter D. A proteomic investigation of similarities between conventional and herbal antidepressant treatments. Journal of Psychopharmacology. 2009;23(5):520–30. 10.1177/0269881108091075 18562437

[76] Pennington K, Beasley C, Dicker P, Fagan A, English J, Pariante C, et al Prominent synaptic and metabolic abnormalities revealed by proteomic analysis of the dorsolateral prefrontal cortex in schizophrenia and bipolar disorder. Molecular psychiatry. 2007;13(12):1102–17. 17938637 10.1038/sj.mp.4002098

[77] Nesvaderani M, Matsumoto I, Sivagnanasundaram S. Anterior hippocampus in schizophrenia pathogenesis: molecular evidence from a proteome study. Australasian Psychiatry. 2009;43(4):310–22. 10.1080/00048670902721103 19296286

[78] Rushlow W, Seah Y, Belliveau D, Rajakumar N. Changes in calcineurin expression induced in the rat brain by the administration of antipsychotics. Journal of neurochemistry. 2005;94(3):587–96. 16033416 10.1111/j.1471-4159.2005.03092.x

